# Anti-Proliferative and Pro-Apoptotic Effects of Licochalcone A through ROS-Mediated Cell Cycle Arrest and Apoptosis in Human Bladder Cancer Cells

**DOI:** 10.3390/ijms20153820

**Published:** 2019-08-05

**Authors:** Su Hyun Hong, Hee-Jae Cha, Hyun Hwang-Bo, Min Yeong Kim, So Young Kim, Seon Yeong Ji, JaeHun Cheong, Cheol Park, Hyesook Lee, Gi-Young Kim, Sung-Kwon Moon, Seok Joong Yun, Young-Chae Chang, Wun-Jae Kim, Yung Hyun Choi

**Affiliations:** 1 Anti-Aging Research Center, Dong-eui University, Busan 47227, Republic of Korea; 2 Department of Biochemistry, Dong-eui University College of Korean Medicine, Busan 47227, Republic of Korea; 3 Department of Parasitology and Genetics, Kosin University College of Medicine, Busan 49267, Republic of Korea; 4 Department of Molecular Biology, College of Natural Sciences, Pusan National University, Busan 46241, Republic of Korea; 5 Department of Molecular Biology, College of Natural Sciences, Dong-eui University, Busan 47340, Republic of Korea; 6 Department of Marine Life Sciences, School of Marine Biomedical Sciences, Jeju National University, Jeju 63243, Republic of Korea; 7 Department of Food and Nutrition, Chung-Ang University, Anseong 17546, Republic of Korea; 8 Department of Urology, College of Medicine, Chungbuk National University, Chungbuk 28644, Republic of Korea; 9 Department of Cell Biology, Catholic University of Daegu School of Medicine, Daegu 42472, Republic of Korea

**Keywords:** Licochalcone A, bladder cancer, G2/M arrest, apoptosis, ROS

## Abstract

Licochalcone A (LCA) is a chalcone that is predominantly found in the root of *Glycyrrhiza* species, which is widely used as an herbal medicine. Although previous studies have reported that LCA has a wide range of pharmacological effects, evidence for the underlying molecular mechanism of its anti-cancer efficacy is still lacking. In this study, we investigated the anti-proliferative effect of LCA on human bladder cancer cells, and found that LCA induced cell cycle arrest at G2/M phase and apoptotic cell death. Our data showed that LCA inhibited the expression of cyclin A, cyclin B1, and Wee1, but increased the expression of cyclin-dependent kinase (Cdk) inhibitor p21WAF1/CIP1, and increased p21 was bound to Cdc2 and Cdk2. LCA activated caspase-8 and -9, which are involved in the initiation of extrinsic and intrinsic apoptosis pathways, respectively, and also increased caspase-3 activity, a typical effect caspase, subsequently leading to poly (ADP-ribose) polymerase cleavage. Additionally, LCA increased the Bax/Bcl-2 ratio, and reduced the integrity of mitochondria, which contributed to the discharge of cytochrome *c* from the mitochondria to the cytoplasm. Moreover, LCA enhanced the intracellular levels of reactive oxygen species (ROS); however, the interruption of ROS generation using ROS scavenger led to escape from LCA-mediated G2/M arrest and apoptosis. Collectively, the present data indicate that LCA can inhibit the proliferation of human bladder cancer cells by inducing ROS-dependent G2/M phase arrest and apoptosis.

## 1. Introduction

Many therapies have been developed for cancer patients, but chemotherapy is still a key therapy in cancer treatment. However, some limitations, such as adverse side effects, drug resistance, and limited efficacy, remain to be overcome [1,2]. Therefore, there is a need to develop more effective and safe treatment options that can minimize these limitations. In this respect, there is increasing attention being paid to the importance of natural compounds derived from herbal medicines and food constituents that have been traditionally used to prevent and treat various diseases [3,4]. In particular, numerous naturally occurring agents have been reported to cause growth arrest and induce apoptosis in cancer cells, without showing toxicity to normal cells [5,6]. These compounds have also emerged as an alternative to chemopreventive and chemotherapeutic agents, because they can specifically regulate various cellular signaling pathways in cancer cells due to enormous structural diversity [7,8]. Moreover, many anti-cancer drugs, including etoposide, vinblastine, paclitaxel, and vincristine, are typical examples of naturally-occurring anti-cancer agents currently in clinical use [9,10,11].

Licochalcone A (LCA, 4’,4-Dihydroxy-3-α,α-dimethylallyl-6-methoxychalcone) is a phenolic chalcone compound found in the root of *Glycyrrhiza glabra* or *G. inflata*, belonging to the plant family Fabaceae, which is widely used in herbal medicines and as a traditional food in Asia [12]. LCA displays a number of pharmacological activities, such as anti-microbial activity [13,14] and anti-inflammatory [15,16] and antioxidant properties [17,18], and is also considered to have potential anti-cancer agents in the results of various cancer cell models. For example, LCA has been reported to inhibit cell proliferation through cell cycle arrest at the G2/M phase in various types of cancer cells, including MCF-7 breast cancer cells [7], A549 lung cancer cells [19], HepG2 human hepatoma cells [20], and U87 glioma cells [21]. In addition, the anti-cancer effects of LCA have been reported in nasopharyngeal cancer, breast cancer, cervical cancer, oral cancer, epithelial ovarian carcinoma, bladder cancer cells, and so on [7,19,20,22,23,24,25,26,27,28,29,30,31,32,33]. These anti-cancer effects have been shown to involve the death receptor (DR)-dependent extrinsic or mitochondria-dependent intrinsic pathways, which are representative apoptosis inducing pathways. It was also found that the anti-cancer effect of LCA was accompanied by the disturbance of various cellular signaling pathways, including mitogen activated protein kinase and phosphatidylinositol 3-kinase/AKT signaling cascades [24,34,35]. Furthermore, LCA showed a strong cytotoxic effect through reactive oxygen species (ROS)-dependent apoptosis in various cancer cell lines [20,25,26,31,32,36,37]. Yuan et al. suggested that LCA induced oxidative stress, and consequently caused T24 cell apoptosis via the mitochondrial-dependent pathway and endoplasmic reticulum (ER) stress [32]. In their subsequent study, they demonstrated that LCA inhibits T24 cell proliferation by increasing intracellular ROS generation [37]. Although they demonstrated that LCA suppresses the proliferation of T24 cells via ROS production, they did not establish an accurate role of ROS. In 2016, the same research team reported that LCA induces apoptosis of T24 bladder cancer cells via increasing intracellular Ca^2+^ levels, which may be associated with mitochondrial dysfunction and ER stress [26]. However, mRNA levels of caspase showed no great change, and caspase activities were not assessed. Although the possibility has recently been proposed of the growth inhibitory activity of LCA in bladder cancer cells [26,32,37], the underlying molecular mechanism remains unclear. Therefore, in this study, we investigated the anti-cancer efficacy of LCA in human bladder cancer cells, focusing on the mechanisms associated with the induction of cell cycle arrest and apoptosis.

## 2. Results

### 2.1. LCA Causes Cell Growth Inhibition in Human Bladder Cancer Cells

**Figure 1:**
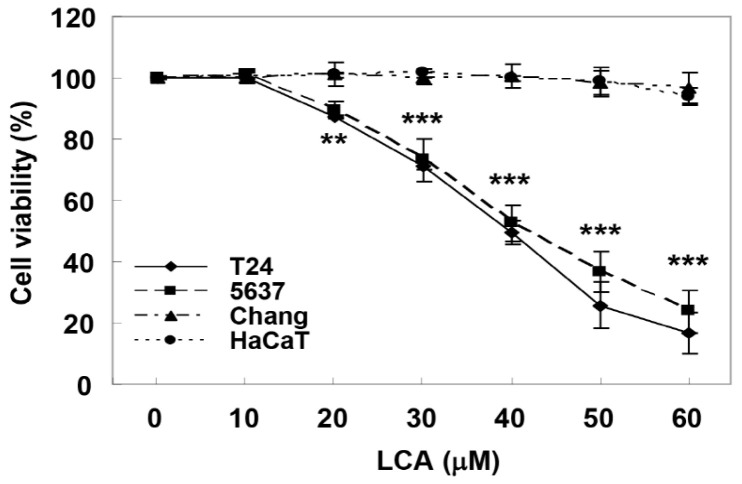
Inhibition of cell proliferation by licochalcone A (LCA) in human bladder cancer cells. T24, 5637, Chang, and HaCat cells were evenly distributed in 96-well plates and treated with LCA (10~60 μM) for 48 h. As described in Materials and Methods, the ability of LCA to inhibit cell proliferation was determined by 3-(4,5-dimethyl-2-thiazolyl)-2,5-diphenyltetra-zolium bromide (MTT) assay, and cell viability values were expressed relative to those wells where no LCA was added (100% control value). Results represent the mean ± standard deviation (SD) of at least three independent experiments (** *p* < 0.001 and *** *p* < 0.0001 compared to control).

To study the anti-cancer effect of LCA in bladder cancer cells, we first determined whether LCA inhibits the proliferation of bladder cancer cells and whether their effects on bladder cancer and normal cells are different. T24 and 5637 cell lines were used as human bladder cancer models, and Chang liver cells and HaCat keratinocytes were used as controls. According to the 3-(4,5-dimethyl-2-thiazolyl)-2,5-diphenyltetra-zolium bromide (MTT) assay results shown in Figure 1, LCA induced a concentration-dependent inhibition of T24 and 5637 cell proliferation. However, no significant inhibition of growth was observed in Chang liver cells and HaCat keratinocytes under the same conditions, indicating that LCA is much more potent at inhibiting the proliferation of human bladder cells than normal cells. In addition, fifty percent inhibitory concentration (IC_50_) values of LCA on T24 and 5637 cells were 40.23 μM and 42.47 μM, respectively. There was no significant difference between T24 and 5637 cells for IC_50_ value, so further experiments were performed on T24 cells. Furthermore, we decided that 40 μM was appropriate as the maximum concentration for further investigation.

### 2.2. LCA Induces G2/M Phase Arrest and Apoptosis in Bladder Cancer T24 Cells

**Figure 2:**
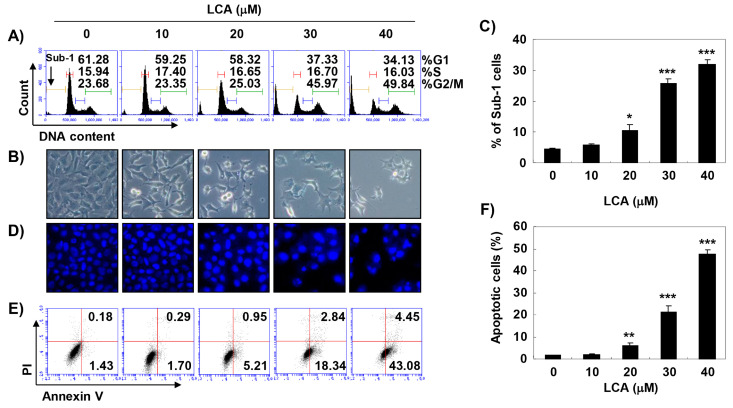
Induction of G2/M arrest and apoptosis by LCA in T24 cells. T24 cells were treated with various concentrations of LCA for 48 h. (**A**,**C**) Cells were stained with propidium iodide (PI) solution for flow cytometry analysis. (**A**) Quantification of the cell population (in percent) in different cell cycle phases of viable cells is shown. (**C**) Sub-G1% was calculated as the percentage of the number of cells in the sub-G1 population relative to the number of total cells. Data were expressed as the mean ± SD of three independent experiments (* *p* < 0.05 and *** *p* < 0.0001 compared to control). (**B**) Morphological changes of T24 cells were observed by phase-contrast microscopy. (**D**) The 4′,6-diamidino-2-phenylindole (DAPI) staining was performed to observe nuclear morphological alterations under an inverted phase-contrast microscope. Representative photographs of the morphological changes are presented. (**E**,**F**) To identify LCA-induced apoptosis, flow cytometry analysis was performed by Annexin V and PI staining. The percentage of annexin V^+^/PI^+^ cells in the top and annexin V^+^/PI^−^ cells in the bottom right quadrant are indicated. Each point represents the mean of three independent experiments. (**E**) Representative profiles. (**F**) The percentages of apoptotic cells were determined by expressing the numbers of Annexin V^+^ cells as percentages of all cells. Each data point represents the mean ± SD of three independent experiments (** *p* < 0.001 and *** *p* < 0.0001 compared to control).

Since LCA can effectively inhibit the growth of human bladder cancer cells, we expected that this inhibitory activity was due to its ability to interfere with cell cycle progression. Therefore, we analyzed cell cycle perturbations after exposure of T24 cells to LCA. Flow cytometry data demonstrated that the percentage of cells arrested at the G2/M phase was increased with increasing LCA treatment concentration, coupled with a decrease in the proportion of cells in G1 and S phases (Figure 2A). Meanwhile, the microscopic examination demonstrated that the phenotypic characteristics of LCA-treated cells showed irregular cell outlines, decreased cell density, cell shrinkage, and increased numbers of detached cells (Figure 2B).

In addition, a significant increase of the cells in the sub-G1 phase, which is used as an index of apoptotic cells, was observed in LCA-treated cells (Figure 2C). Therefore, 4′,6-diamidino-2-phenylindole (DAPI) staining was performed to investigate whether apoptosis was involved in cell growth inhibition induced by LCA. Figure 2D indicates that morphological changes of the nuclei observed in cells undergoing apoptosis, such as nuclear fragmentation and chromatin condensation, were commonly found in LCA-treated T24 cells. To quantify the apoptosis triggered by LCA, annexin V-fluorescein isothiocyanate (FITC)/propidium iodide (PI) double staining assay was conducted. As shown in Figure 2E,F, the results of the flow cytometric analysis showed that the percentage of annexin V^+^/PI^−^ cells and annexin V^+^/PI^+^ cells was markedly increased in LCA-treated cells in a dose-dependent manner. Taken together, these results indicate that LCA-induced G2/M phase arrest was associated with the induction of apoptosis. 

### 2.3. LCA Regulates the Expression of G2/M Phase-Associated Proteins in T24 Cells

**Figure 3:**
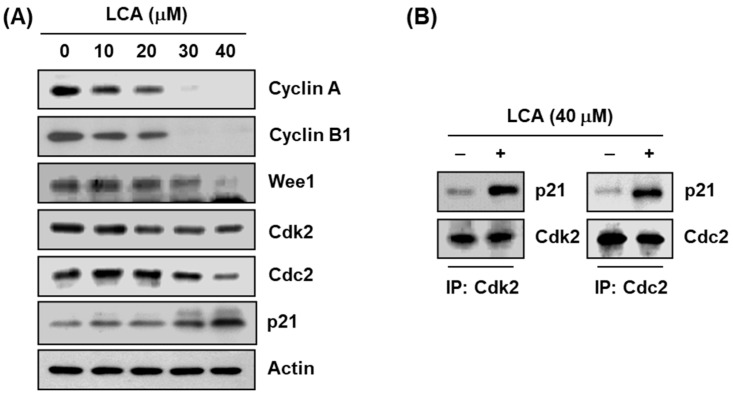
Effects of LCA on the levels of cell cycle regulatory genes in T24 cells. (**A**) After treatment with LCA for 48 h, total cell lysates were prepared. Equal amounts of cellular proteins were separated in sodium dodecyl sulfate (SDS)-polyacrylamide gels, and transferred to polyvinylidene difluoride (PVDF) membranes. The membranes were probed with the indicated antibodies, and the proteins were visualized using an enhanced chemiluminescence (ECL) detection system. Actin was used as an internal control for Western blot assays. (**B**) Cells were incubated without or with 40 μM LCA for 48 h, and then equal amounts of proteins were immunoprecipitated with the anti-Cdc2 or Cdk2 antibody. Western blotting using immunocomplexes was performed using anti-p21, Cdc2, or Cdk2 antibody, and an ECL detection system. Note: IP = immunoprecipitation.

To explore the biochemical events of LCA-elicited cell cycle arrest, levels of G2/M phase-associated proteins were analyzed. Immunoblotting results revealed that following LCA treatment, the levels of cyclin A, cyclin B1, and Wee1 were reduced, and the effect was concentration dependent, while the expression of cyclin-dependent kinase (Cdk) 2 and cell division cycle (Cdc) 2 was relatively maintained at the level of the control group (Figure 3A). However, the expression of p21WAF1/CIP1, a Cdk inhibitor, was markedly induced in response to LCA exposure. Additionally, we performed co-immunoprecipitation to investigate the role of LCA-induced p21, and found that increased p21 by LCA-treated cells was complexed with Cdk2 and Cdc2 (Figure 2B). 

### 2.4. LCA Activates Caspases in T24 Cells

**Figure 4:**
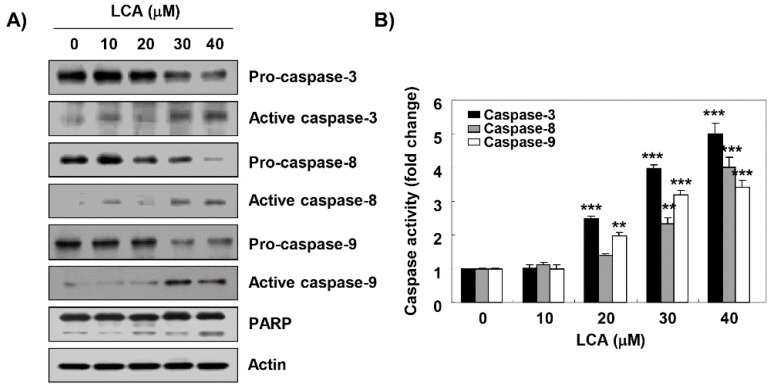
Activation of caspases and degradation of poly (ADP-ribose) polymerase (PARP) by LCA in T24 cells. T24 cells were treated with the indicated concentrations of LCA for 48 h. (**A**) Equal amounts of cellular proteins were separated in SDS-polyacrylamide gels, and transferred to PVDF membranes. Membranes were probed with the indicated antibodies, and proteins were visualized (**B**). The activities of caspases were evaluated using caspase colorimetric assay kits. Each data point represents the mean ± SD of three independent experiments (** *p* < 0.001 and *** *p* < 0.0001, when compared to control).

We next assessed whether LCA activated the caspase signaling pathway in order to examine the pathway of LCA-induced apoptosis. Our results showed that LCA decreased the expression of pro-caspase-8, -9, and -3, but increased the expression of their active forms, which was associated with the degradation of poly(ADP-ribose) polymerase (PARP), an effector substrate of caspase-3. Therefore, we quantitatively assessed each caspase activity in the presence of LCA using fluorogenic substrates to determine whether these immunoblotting results were directly related to activation of the corresponding caspases, and found that treatment with LCA significantly stimulated the activation of these caspases in a concentration-dependent manner, in comparison with untreated control cells (Figure 4B). 

### 2.5. LCA Increases Mitochondrial Dysfunction in T24 Cells

**Figure 5:**
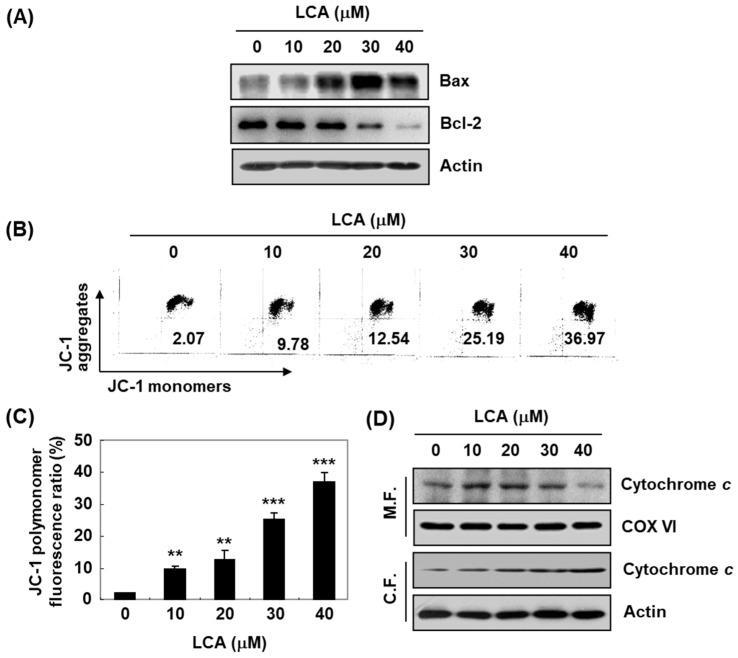
Effects of LCA on the values of mitochondrial membrane potential (MMP), and expression of Bcl-2 family members and cytochrome c in T24 cells. T24 cells were treated with different concentrations of LCA for 48 h. (**A**) Cell lysates were prepared, and Western blotting was then per-formed using the indicated antibodies. (**B**,**C**) Cells were collected and stained with 5,5‘,6,6’-tetrachloro-1,1’,3,3’-tetraethyl-imidacarbocyanine iodide (JC-1) dye, and were then analyzed by a flow cytometer to evaluate the changes in MMP. (**B**) Representative profiles. (**C**) Each bar represents the percentage of cells with JC-1 aggregates (mean ± SD of triplicate determinations, ** *p* < 0.001, *** *p* < 0.0001, when compared to control). (**D**) Cytosolic and mitochondrial proteins were prepared and analyzed for cytochrome c expression by Western blot analysis. Equal protein loading was confirmed by the analysis of cytochrome oxidase subunit VI (COX VI) and actin in each protein extract. Note: M.F. = mitochondrial fraction; C.F. = cytoplasmic fraction.

The effects of LCA on the expression of Bcl-2 family members, which play a critical role in determining the induction of apoptosis, were also determined. As shown in Figure 5A, expression of pro-apoptotic Bax protein was increased, while the levels of anti-apoptotic Bcl-2 protein were reduced in LCA-stimulated cells. We further assessed whether mitochondrial dysfunction was involved in LCA-induced apoptosis. According to flow cytometry results using 5,5‘,6,6’-tetrachloro-1,1’,3,3’-tetraethyl-imidacarbocyanine iodide (JC-1), the mitochondrial membrane potential (MMP)-dependent formation of JC-1 aggregates in mitochondria was maintained at a relatively high rate in T24 cells not treated with LCA. However, the proportion of JC-1 aggregates was significantly decreased with increasing concentrations of LCA treatment (Figure 5B,C), indicating a significant depletion of MMP after LCA treatment. Additionally, cytochrome *c* was markedly accumulated in the cytoplasm in the LCA-treated cells, but decreased in the mitochondrial fraction (Figure 5D).

### 2.6. LCA Induces ROS-Dependent Mitochondrial Dysfunction in T24 Cells

**Figure 6:**
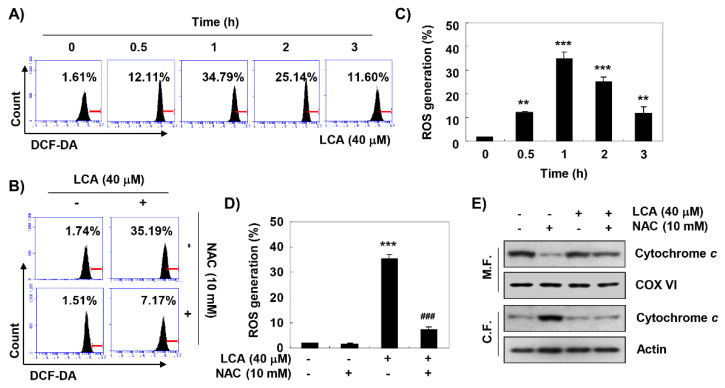
Accumulation of reactive oxygen species (ROS) by LCA in T24 cells. (**A**,**B**) The cells were pre-treated with or without 10 mM N-acetyl-L-cysteine (NAC) for 1 h before LCA treatment. ROS generation was measured by a flow cytometer using DCF-DA dye. (**C**,**D**) Each bar represents the mean ± SD of three independent experiments (** *p* < 0.001 and *** *p* < 0.0001 compared to control; ^###^ *p* < 0.0001 compared to LCA-treated cells). (**E**) The cells were treated with 40 μM LCA for 48 h or pre-treated with 10 mM NAC for 1 h before 40 μM LCA treatment. The cell lysates were prepared, and the expression of cytochrome *c* protein was evaluated by Western blot analysis.

Flow cytometry analysis using 5,6-carboxy-2′,7′-dichlorodihydrofluorescein diacetate (DCF-DA) was performed to assess the involvement of ROS in LCA-induced mitochondrial dysfunction. Our data indicated that the accumulation of ROS peaked at 1 h of LCA treatment, and then gradually decreased (Figure 6A,C). However, cells pre-treated with N-acetyl-L-cysteine (NAC), a potent ROS scavenger, showed a significant decrease in ROS levels compared to cells treated with LCA alone (Figure 6B,D). In addition, when the production of ROS was artificially blocked, the reduced levels of cytochrome *c* in mitochondria by LCA were attenuated (Figure 6E). 

### 2.7. ROS Acts as an Upstream Regulator of LCA-Induced Growth Arrest and Apoptosis in T24 Cells

**Figure 7:**
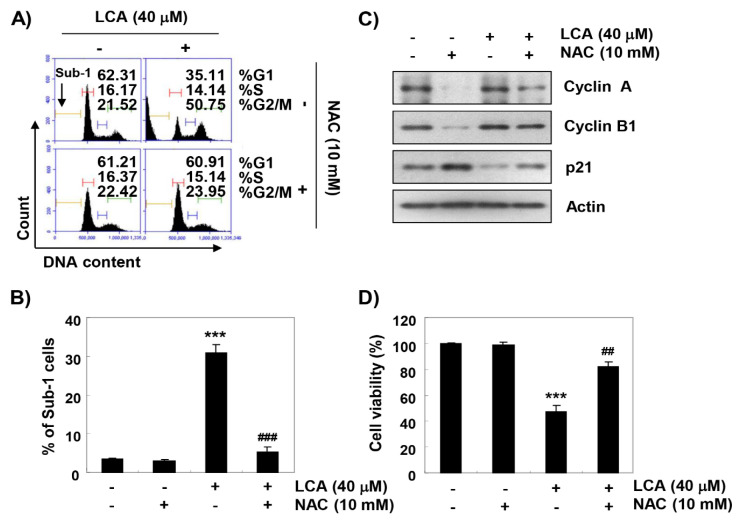
Roles of ROS on LCA-induced cell cycle arrest and apoptosis in T24 cells. The cells were either treated with 40 μM LCA for 48 h or pre-treated with 10 mM NAC for 1 h before 40 μM LCA treatment and then collected. (**A**,**B**) Cells were stained with PI solution for flow cytometry analysis. (**A**) Representative profiles. Quantification of the cell population (in percent) in different cell cycle phases of viable cells is shown. (**B**) The percentages of apoptotic sub-G1 were calculated as the percentage of the number of cells in the sub-G1 population, relative to the number of total cells. (**C**) Expression of cyclin A, cyclin B1, and p21 protein was evaluated by Western blot analysis. (**D**) The cell viability was determined by MTT assay. Each bar represents the mean ± SD of three independent experiments (*** *p* < 0.0001 compared to control; *^##^* *p* < 0.001 compared to LCA-treated cells; ^###^ *p* < 0.0001 compared to LCA-treated cells).

The effect of ROS on LCA-mediated G2/M arrest and apoptosis was further investigated to determine the role of ROS in the anti-cancer activity of LCA in T24 cells. As depicted in the results of flow cytometry analysis, blockade of ROS generation reversed LCA-induced cell cycle arrest at the G2/M stage (Figure 7A), which was associated with a reduction in the frequency of sub-G1 phase cells (Figure 7B). Furthermore, the presence of NAC effectively prevented the LCA-induced decrease of cyclin A and cyclin B1, and markedly abolished the enhanced expression of p21 by LCA (Figure 7C). In parallel with these results, inhibiting ROS production significantly attenuated LCA-induced cell death (Figure 7D), demonstrating the contribution of ROS generation in the G2/M arrest and apoptosis observed in LCA-treated T24 cells. 

## 3. Discussion

Disruption of cell cycle regulation is clearly implicated in the development and progression of most tumors, and discontinuation of this process is considered to be an important strategy to inhibit the proliferation of cancer cells [5,6]. In particular, it is clear that the induction of apoptosis by many anti-cancer agents is associated with cell cycle arrest at specific checkpoints [38,39]. Therefore, we first investigated whether the suppression of bladder cancer cell proliferation by LCA was associated with cell cycle arrest. The results of flow cytometry analysis showed that LCA caused G2/M phase arrest in the T24 human bladder cancer cell line, similar to the results of previous studies in several human cancer cell lines [7,19,20,21,22], suggesting that G2/M phase arrest is one of the mechanisms of the anti-cancer effect of LCA in T24 cells. 

The progression of the cell cycle in eukaryotic cells is strictly controlled by the interaction of cyclins and Cdks with their inhibitory factors. For example, D-type cyclins bind to and activate Cdk4 and Cdk6, leading to sequential progression from the G1 to S phase. On the other hand, cyclin A/Cdk2 and Cdc2 complexes control S and G2 phases, while cyclin B/Cdc2 complex regulates induction of G2/M transition and achievement of mitosis [38,40]. In addition, Wee1, a tyrosine kinase, induces phosphorylation of Cdc2, resulting in inhibition of cyclin B-Cdc2 activity and preventing entry into mitosis [41,42]. In the current study, when T24 cells were exposed to LCA, expression of cyclin A, cyclin B1, and Wee1 was markedly reduced without significant changes in expression of Cdk2 and Cdc2. Consistent with the results of previous studies reported in human hepatoma and oral squamous cell carcinoma cells [20,30], we also found that expression of p21 in LCA-treated T24 cells was significantly induced. The typical Cdk inhibitor p21, belonging to the KIP/CIP family, was first reported as a major inducer of tumor suppressor p53-dependent cell cycle arrest induced by DNA damage, but it can act as a mediator of p53-independent cell arrest in various types of cancer cells [43,44]. When p21 expression increases, it forms complexes with Cdks to reduce kinase activity and inhibit cell cycle progression [43,45]. Our data showed that complexes immunoprecipitated with Cdk2 and Cdc2 antibodies in LCA-treated cells contained a greater amount of immunologically detectable p21 protein compared to untreated control cells, which may have contributed the reduction of Cdk2 and Cdc2 activity, ultimately leading to G2/M arrest. Because T24 cells are mutant p53 gene-bearing cells [46], increased p21 expression by LCA seems to contribute to G2/M inhibition regardless of p53 gene status. Collectively, our data suggest that LCA-triggered G2/M arrest was due to decreased expression of cyclin A, cyclin B1, and Wee1, and an increase in p21 levels through a p53-independent mechanism.

Since LCA-mediated cell cycle arrest at the G2/M phase has been reported to be related to apoptosis in various cancer cell lines [7,19,20,22], we also evaluated whether G2/M arrest by LCA was associated with apoptosis induction in T24 cells. Based on the results of morphological changes and flow cytometry analysis, we found that the anti-proliferative effect of LCA was achieved through the induction of apoptosis associated with G2/M arrest. Apoptosis is a highly conserved physiological mechanism for eliminating malignant cells without causing damage to normal cells or surrounding tissues, and can be broadly divided into extrinsic and intrinsic pathways in mammalian cells [47,48]. The extrinsic pathway is characterized by the activation of caspase-8 by the formation of the death-inducing signal complex through binding of death ligands to the cell surface DRs [48,49]. On the other hand, activation of the intrinsic pathway is caused by the activation of caspase-9 through the release of pro-apoptotic factors, such as cytochrome *c*, from the mitochondrial intermembrane space into the cytoplasm. This pathway is tightly regulated by the Bcl-2 protein family that includes pro- and anti-apoptotic proteins, which guard mitochondrial integrity, and control the release of cytochrome *c* through the mitochondrial transition pore [47,50]. Caspases-8 and -9, which correspond to the initiator caspases of each pathway, ultimately activate apoptosis through the cleavage of various cellular substrates, such as PARP, by activating down stream executioner caspases, including caspase-3 and -7 [47,48,49,50]. As shown in previous studies using human nasopharyngeal and ovarian carcinoma cells [23,33], our results demonstrate that LCA activated caspase-3 as well as caspase-8 and -9, and induced the cleavage of PARP. Therefore, we speculated that the pro-apoptotic effect of LCA in T24 cells could occur by simultaneously activating extrinsic and intrinsic pathways. In addition, consistent with previous studies [24,25,26,27,28,32], we confirmed that mitochondrial dysfunction was induced in LCA-treated cells, which was accompanied by the promotion of cytosolic release of cytochrome *c*, suggesting that cytochrome *c* release was due to an increase in mitochondrial membrane permeability. Additionally, we examined the effects of LCA on the expression of Bax and Bcl-2, both of which regulate mitochondrial membrane permeabilization [47,50], and found that LCA down-regulated Bcl-2/Bax ratio. These results imply that LCA treatment shifted the equilibrium between Bax and Bcl-2 in mitochondria, resulting in permeability of the mitochondrial membrane.

Increasing evidence demonstrates that many anti-cancer agents promote growth inhibition and apoptosis for removal of cancer cells through pro-oxidant properties, such as increasing ROS accumulation, or destroying cellular antioxidant systems [51,52]. Several previous studies have reported that many phytochemicals generate ROS to activate cell cycle arrest and apoptosis signaling in cancer cells [53,54,55]. In particular, mitochondria are a major target of ROS and a major subcellular organelle responsible for the production of ROS in the cells [56,57]. These observations suggest that inducing the production of ROS in cancer cells can be used in therapeutic strategies. Therefore, we further assessed whether LCA-induced G2/M arrest and apoptosis in T24 cells was correlated with the production of ROS. Consistent with previous studies [20,25,26,31,32,36], our results showed that LCA-treatment markedly increased the levels of ROS production. However, the quenching of ROS generation using NAC significantly diminished LCA-induced cytosolic release of cytochrome *c* to control levels, indicating that ROS act as upstream signaling molecules to enhance LCA-mediated mitochondrial dysfunction in T24 cells. Our results also demonstrated that the presence of NAC markedly attenuated LCA-induced down-regulation of cyclin A and cyclin B1, and up-regulation of p21. Subsequently, NAC pre-treatment also significantly protected against LCA-mediated G2/M arrest and cell death, confirming that increasing ROS may serve a key contributor to the anti-cancer effects of LCA.

Taken together, these results suggest that the production of ROS by LCA plays a critical role in the induction of G2/M arrest and apoptosis through simultaneous initiation of both extrinsic and intrinsic pathways in human bladder cancer cells. However, future studies are warranted to explore the precise molecular mechanisms of intracellular signaling pathways associated with the production of ROS and to evaluate the anti-cancer properties of LCA in in vivo bladder cancer models. In addition, further studies are required for bioavailability and PK analysis for clinical application of LCA. These further studies are expected to suggest the scientific evidence of LCA for treatment or prevention of cancers, including bladder cancer.

## 4. Materials and Methods

### 4.1. Cell Culture and LCA Treatment

Human bladder cancer T24 and 5637, Chang liver, and HaCat keratinocyte cells were purchased from the American Type Culture Collection (Manassas, MD, USA). T24 and 5637 cells were cultured in RPMI 1640 medium containing 10% heat-inactivated fetal bovine serum (FBS). Chang and HaCaT cells were cultured in Eagle’s minimum essential medium and Dulbecco’s modified Eagle’s medium, respectively, containing 10% FBS. All media were also supplemented with 100 U/mL of penicillin and 100 μg/mL of streptomycin (all from WelGENE Inc., Daegu, Korea) at 37 °C in an atmosphere containing 5% CO_2_ in a humidified incubator. LCA obtained from Sigma-Aldrich Chemical Co. (St. Louis, MO, USA) was dissolved in dimethyl sulfoxide (DMSO, Sigma-Aldrich Chemical Co., St. Louis, MO, USA) as a 100 mM stock solution.

### 4.2. Cell Viability

The cells were seeded at a density of 1 × 10^4^ cells/well in 96-well plates and allowed to attach to the well. After 24 h, cells were treated with the desired concentrations of LCA in triplicate for 48 h and the cells were then incubated with 50 μg/mL MTT solution (Invitrogen, Waltham, MA, USA) for 2 h. The formazan crystals were dissolved in DMSO, and the optical density of the solution was determined using a microplate reader (Molecular Device Co., Sunnyvale, CA, USA) at 540 nm wavelength to determine the number of viable cells. The morphological changes of cells were observed and photographed under an inverted phase-contrast microscope (Carl Zeiss, Oberkochen, Germany).

### 4.3. Determination of Cell Cycle Distribution by Flow Cytometric Analysis

PI staining was applied to analyze the DNA content and cell cycle distribution. After treatment with different concentrations of LCA for 48 h, the detached cells and adherent cells were collected, and cell suspensions of approximately 1 × 10^6^ cells were generated. The cells were fixed in ice-cold 70% ethanol (in phosphate-buffered saline, PBS) at 4 °C for 30 min. The cells were re-suspended in PBS containing 40 μg/mL PI, 100 μg/mL RNase A, 0.1% (*w*/*v*) sodium citrate, and 0.1% (*v*/*v*) Nonidet-P40 (all from Sigma-Aldrich Chemical Co.), then incubated on ice for 30 min in the dark at room temperature. Finally, the cells were analyzed for cell cycle perturbation using a flow cytometer (BD Biosciences, San Jose, CA, USA) and Cell Quest software was used to determine the relative DNA content. The frequency of sub-G1 cells was calculated to estimate apoptotic cells.

### 4.4. Detection of Apoptotic Morphological Changes

Changes of nuclear morphology were assessed by DAPI staining. After 48 h treatment with LCA, the cells were washed with PBS, and fixed with 3.7% paraformaldehyde (Sigma-Aldrich Chemical Co.) in PBS for 10 min at room temperature. The fixed cells were washed with PBS, and stained with 1 μg/mL DAPI solution (Sigma-Aldrich Chemical Co.) for 10 min in the dark. The cells were washed with PBS, and the fluorescence intensity was observed and photographed under a fluorescence microscope (Carl Zeiss, Oberkochen, Germany).

### 4.5. Determination of Apoptotic Cell Death by Flow Cytometric Analysis

Apoptotic cell death was quantified by a flow cytometer using the Annexin V Apoptosis Detection Kit from BD Biosciences (San Diego, CA, USA), according to the manufacturer’s instruction. In brief, following treatment of the cells with LCA for 48 h, the detached cells and adherent cells were collected. The collected cells were suspended in the supplied binding buffer and then stained with FITC-conjugated annexin V and PI at room temperature in the dark for 20 min. The cells were resuspended in binding buffer and analyzed using a flow cytometer, and the annexin V^+^/PI^−^ and annexin V^+^/PI^+^ cell populations were considered indicators of apoptotic cells.

### 4.6. Protein Extraction, Co-Iimmunoprecipitation, and Western Blot Analysis

Whole cell protein extracts were prepared and protein concentrations measured using Bradford protein assay kit (Bio-Rad Laboratories, Hercules, CA, USA), according to the manufacturer’s instructions. For co-immunoprecipitation assays, 500 μg of cell lysates from each sample was precleaned with normal rabbit IgG and protein-A-sepharose bead slurry (Amersham, Arlington Heights, IL, USA), and immunoprecipitation was conducted using 1 μg of anti-Cdk2 or Cdc2 antibody (Santa Cruz Biotechnology, Inc., Santa Cruz, CA, USA) and protein-A-sepharose (Sigma-Aldrich Chemical Co.). Protein complexes were then prepared according to previously described methods [58]. Equal amounts of protein samples or immunoprecipitated proteins were separated by denaturing SDS-polyacrylamide gel electrophoresis, and then transferred onto PVDF membranes (Millipore, Bedford, MA, USA). The membranes were blocked with 5% skim milk in Tris-buffered saline containing 0.1% Triton X-100 (TBST) for 1 h at room temperatue, and probed with specific primary antibodies (Santa Cruz Biotechnology, Inc. and Cell Signaling Technology) at 4 °C overnight. After washing with TBST, the membranes were incubated with the appropriate horseradish peroxidase-conjugated secondary antibodies (Santa Cruz Biotechnology, Inc.) for 2 h. The expression of protein was detected by ECL kit (GE Healthcare Life Sciences, Little Chalfont, UK), and visualized by Fusion FX Image system (Vilber Lourmat, Torcy, France). All results were confirmed in three independent experiments.

### 4.7. Caspase Activity Assay

The activity of caspases was determined using Caspase Activity Assay Kits (R&D Systems, Minneapolis, MN, USA), according to the protocol of the manufacturer. Briefly, the detached cells and adherent cells were harvested, washed with PBS, and cell pellets resuspended in the lysis buffer provided in the kit. The supernatants were collected and incubated with the supplied reaction buffer containing dithiothreitol, with or without tetrapeptides labeled with p-nitroaniline (pNA) at 37 °C for 2 h under light-shielded conditions. The caspase activities were determined by measuring changes in absorbance at 405 nm using a microplate reader.

### 4.8. Measurement of MMP (ΔΨm) and ROS Production

To measure MMP, JC-1 staining was performed. After LCA treatment for 48 h, 10 μM JC-1 (Sigma-Aldrich Chemical Co.) was added to the cells for 30 min at 37 °C. Subsequently, the cells were washed with PBS to remove unbound dye, and at least 10,000 cells were collected for each sample. The amounts of MMP were detected at 488/575 nm using a flow cytometer by following the manufacturer’s protocol [59]. Briefly, the gated region of the y-axis (JC-1 aggregate) included cells with intact mitochondrial membranes and the gated region of the x-axis (JC-1 monomer) depicted cells with loss of MMP. The production of ROS was measured using DCF-DA. At the end of the treatment with LCA, 10 μM DCF-DA (Invitrogen) was added to the incubated cells in a dark environment for 20 min at 37 °C. Subsequently, cells were analyzed for DCF fluorescence by a flow cytometer at 480 nm/520 nm.

### 4.9. Statistical Analysis

The results of quantitative studies are reported as mean ± SD using GraphPad Prism software (version 5.03; GraphPad Software, Inc., La Jolla, CA, USA). All experiments were repeated at least three times. To compare data, one-way analysis of variance (ANOVA) with Dunnett’s post hoc test was used, and *p* < 0.05 was considered to indicate a statistically significant difference.

## 5. Conclusions

**Figure 8:**
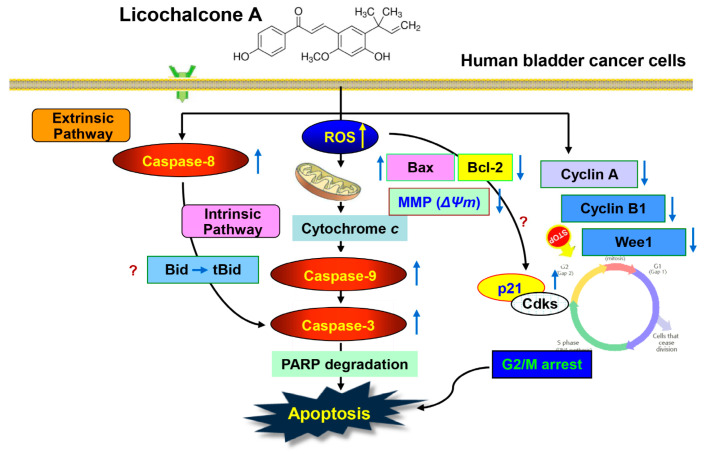
A schematic diagram of LCA-elicited cell cycle arrest and apoptosis in T24 human bladder cancer cells established in this study. ↑: up-regulation; ↓: down-regulation.

We provide evidenced here that LCA exerts an anti-proliferative effect on human bladder cancer cells through the induction of cell cycle arrest at G2/M phase (Figure 8). LCA-induced G2/M arrest was attributed to the decrease in cyclin A, cyclin B1, and Wee1 expression and the increase of p21. LCA also induced apoptosis by leading to the degradation of PARP through activation of effector caspase-3 that results from activating caspase-8 and -9, which belong to the initiator caspases of the extrinsic and intrinsic pathways, respectively. In addition, LCA enhanced the mitochondrial dysfunction, which was associated with an increase in Bax/Bcl-2 expression ratio and the release of cytochrome *c* from the mitochondria into the cytosol. Moreover, the induction of G2/M arrest and apoptosis by LCA was accompanied by the excessive production of ROS. However, the interruption of ROS generation led cells to escape from G2/M arrest and apoptosis. Based on these findings, we suggest that LCA has bladder cancer treatment potential by ROS-dependent induction of G2/M arrest and apoptosis in T24 cells.

## Abbreviations

Cdc	Cell division cycle
Cdk	Cyclin-dependent kinase
COX VI	Cytochrome oxidase subunit VI
DAPI	4′,6-diamidino-2-phenylindole
DCF-DA	5,6-carboxy-2′,7′-dichlorodihydrofluorescein diacetate
DMSO	Dimethyl sulfoxide
DR	Ddeath receptor
ECL	Enhanced chemiluminescence
FBS	Fetal bovine serum
FITC	Fluorescein isothiocyanate
IP	Immunoprecipitation
JC-1	5,5‘,6,6’-tetrachloro-1,1’,3,3’-tetraethyl-imidacarbocyanine iodide
LCA	Licochalcone A
MMP	Mitochondrial membrane potential
MTT	3-(4,5-dimethyl-2-thiazolyl)-2,5-diphenyltetra-zolium bromide
NAC	N-acetyl-L-cysteine
p-NA	P-nitroaniline
PARP	Poly(ADP-ribose) polymerase
PBS	Phosphate-buffered saline
PI	Propidium iodide
PVDF	Polyvinylidene difluoride
ROS	Reactive oxygen species
SD	Standard deviation
SDS	Sodium dodecyl sulfate
