# Correction: Hong et al. Anti-Proliferative and Pro-Apoptotic Effects of Licochalcone A through ROS-Mediated Cell Cycle Arrest and Apoptosis in Human Bladder Cancer Cells. *Int. J. Mol. Sci.* 2019, *20*, 3820

**DOI:** 10.3390/ijms252413595

**Published:** 2024-12-19

**Authors:** Su Hyun Hong, Hee-Jae Cha, Hyun Hwang-Bo, Min Yeong Kim, So Young Kim, Seon Yeong Ji, JaeHun Cheong, Cheol Park, Hyesook Lee, Gi-Young Kim, Sung-Kwon Moon, Seok Joong Yun, Young-Chae Chang, Wun-Jae Kim, Yung Hyun Choi

**Affiliations:** 1 Anti-Aging Research Center, Dong-eui University, Busan 47227, Republic of Korea; hongsh@deu.ac.kr (S.H.H.); hhyun@deu.ac.kr (H.H.-B.); ksso14@naver.com (M.Y.K.); 14731@deu.ac.kr (S.Y.K.); 14602@deu.ac.kr (S.Y.J.); lhyes0219@pusan.ac.kr (H.L.); 2 Department of Biochemistry, Dong-eui University College of Korean Medicine, Busan 47227, Republic of Korea; 3 Department of Parasitology and Genetics, Kosin University College of Medicine, Busan 49267, Republic of Korea; hcha@kosin.ac.kr; 4 Department of Molecular Biology, College of Natural Sciences, Pusan National University, Busan 46241, Republic of Korea; molecule85@pusan.ac.kr; 5 Department of Molecular Biology, College of Natural Sciences, Dong-eui University, Busan 47340, Republic of Korea; parkch@deu.ac.kr; 6 Department of Marine Life Sciences, School of Marine Biomedical Sciences, Jeju National University, Jeju 63243, Republic of Korea; immunkim@jejunu.ac.kr; 7 Department of Food and Nutrition, Chung-Ang University, Anseong 17546, Republic of Korea; sumoon66@cau.ac.kr; 8 Department of Urology, College of Medicine, Chungbuk National University, Chungbuk 28644, Republic of Korea; sjyun@chungbuk.ac.kr; 9 Department of Cell Biology, Catholic University of Daegu School of Medicine, Daegu 42472, Republic of Korea; ycchang@cu.ac.kr

In the original publication [1], there was a mistake in Figures 3A and 5A as published. Figures 3A and 5A contain Western blotting image errors. In Figures 3A and 5A, Western blotting image for p21 and Bcl-2 are recognized as the same image with left and right side rotated. The error was recently recognized and verified, and we would like to correct the error. The corrected Figure 3A and Figure 5A appear below. The authors state that the scientific conclusions are unaffected. This correction was approved by the Academic Editor. The original publication has also been updated.

## Figures and Tables

**Figure 3:**
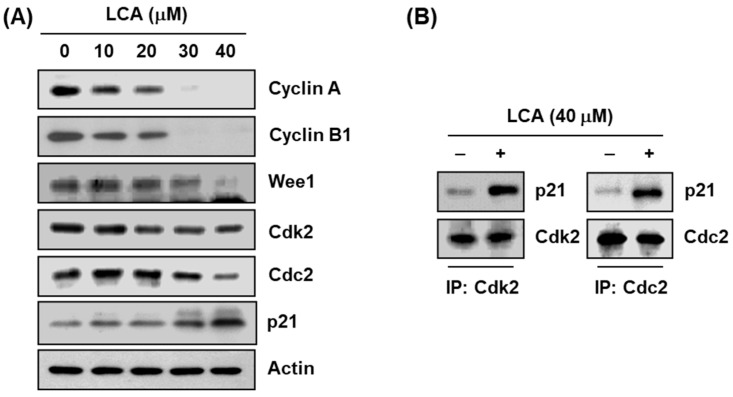
Effects of LCA on the levels of cell cycle regulatory genes in T24 cells. (**A**) After treatment with LCA for 48 h, total cell lysates were prepared. Equal amounts of cellular proteins were separated in sodium dodecyl sulfate (SDS)-polyacrylamide gels, and transferred to polyvinylidene difluoride (PVDF) membranes. The membranes were probed with the indicated antibodies, and the proteins were visualized using an enhanced chemiluminescence (ECL) detection system. Actin was used as an internal control for Western blot assays. (**B**) Cells were incubated without or with 40 μM LCA for 48 h, and then equal amounts of proteins were immunoprecipitated with the anti-Cdc2 or Cdk2 antibody. Western blotting using immunocomplexes was performed using anti-p21, Cdc2, or Cdk2 antibody, and an ECL detection system. Note: IP = immunoprecipitation.

**Figure 5:**
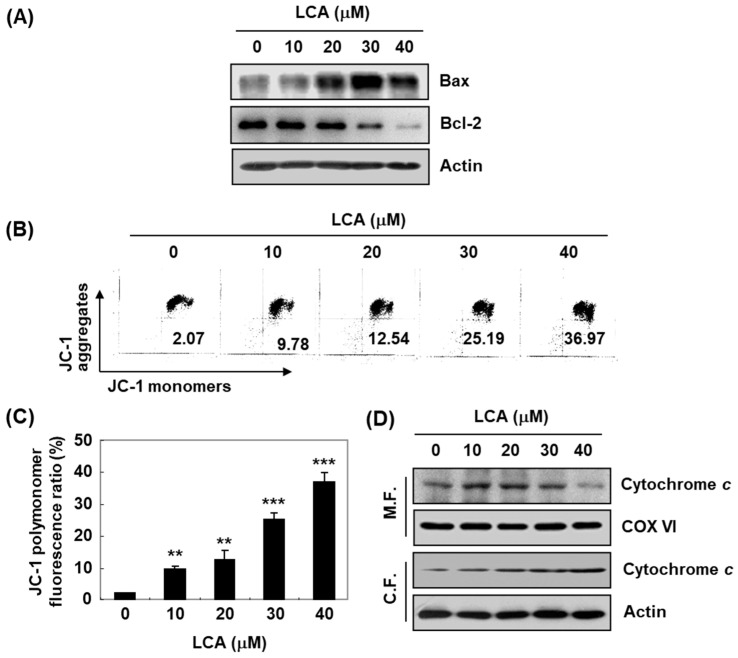
Effects of LCA on the values of mitochondrial membrane potential (MMP), and expression of Bcl-2 family members and cytochrome c in T24 cells. T24 cells were treated with different concentrations of LCA for 48 h. (**A**) Cell lysates were prepared, and Western blotting was then per-formed using the indicated antibodies. (**B**,**C**) Cells were collected and stained with 5,5‘,6,6’-tetrachloro-1,1’,3,3’-tetraethyl-imidacarbocyanine iodide (JC-1) dye, and were then analyzed by a flow cytometer to evaluate the changes in MMP. (**B**) Representative profiles. (**C**) Each bar represents the percentage of cells with JC-1 aggregates (mean ± SD of triplicate determinations, ** *p* < 0.001, *** *p* < 0.0001, when compared to control). (**D**) Cytosolic and mitochondrial proteins were prepared and analyzed for cytochrome c expression by Western blot analysis. Equal protein loading was confirmed by the analysis of cytochrome oxidase subunit VI (COX VI) and actin in each protein extract. Note: M.F. = mitochondrial fraction; C.F. = cytoplasmic fraction.
